# Study on the Influence of Opposing Glare from Vehicle High-Beam Headlights Based on Drivers’ Visual Requirements

**DOI:** 10.3390/ijerph19052766

**Published:** 2022-02-27

**Authors:** Jiangbi Hu, Yunpeng Guo, Ronghua Wang, Sen Ma, Aolin Yu

**Affiliations:** Faculty of Architecture, Civil and Transportation Engineering, Beijing University of Technology, Beijing 100124, China; hujiangbi@bjut.edu.cn (J.H.); guoyunpeng@emails.bjut.edu.cn (Y.G.); masen@emails.bjut.edu.cn (S.M.); yal_bjut@163.com (A.Y.)

**Keywords:** anti-glare facility, traffic glare, high-beam headlight glare, illuminance thresholds, spatial distribution

## Abstract

The anti-glare facilities in median strips are designed to block opposing headlights in order to avoid disability glare, but a large amount of headlight leakage results in uncomfortable glare, to the point that drivers can barely detect dangerous obstacles or road conditions. This paper aims to explore the glare range under high-beam headlights on drivers’ visual requirements. Based on an analysis of the mechanism of headlight glare, this paper proposes a subjective headlight glare scale, and classifies glare discomfort into two categories: interference glare, and acceptability glare. Combining the scales, 24 drivers and a standard light-emitting diode automotive headlamp were used to conduct glare effect tests. The size of the laboratory that closes to scotopic vision is 12 m × 6 m. The illuminance thresholds of disability glare–interference glare (DGIG) and interference glare–acceptability glare (IGAG), along with the spatial distribution of each glare level, were collected at the longitudinal distances of 3 m, 5 m, 7 m, 10 m, and 12 m. Meanwhile, the illuminance threshold and the spatial distribution of each glare level up to a longitudinal distance of 120 m were calculated. The results indicate that disability glare is distributed in the central area, while interference glare and acceptability glare are distributed from the center to the margins. At the same longitudinal distance, the vertical illuminance of the driver’s eye under the same glare level is almost equal. In the range of a longitudinal distance of 120 m, the spatial distribution of each glare level enlarges with each increase in longitudinal distance. The results can provide scientific evidence for calculating the reasonable heights of anti-glare facilities for expressways with different alignments.

## 1. Introduction

Glare is the condition of vision in which there is discomfort or a reduction in the ability to see details or objects, caused by an unsuitable distribution or range of luminance, or by extreme luminance contrasts [1]. Glare from high-beam headlights is an adverse factor that affects the acquisition of visual information for oncoming drivers at night. Some studies [2,3,4,5,6] have shown that glare caused by oncoming vehicles can reduce the visual ability, judgment, and response ability of pedestrians and drivers. According to statistics, half of the fatal accidents on U.S. roads occur at night, and the number of traffic accidents caused by glare from the high-beam headlights of oncoming traffic at night accounts for 12% to 15% of all traffic accidents [7]. The installation of anti-glare facilities in the highway median strip is one of the most effective ways to block the glare from oncoming vehicles. Fernandes [8] and Hammond et al. [9] considered installing anti-glare facilities, which is one of the essential methods to solve the problem of high-beam headlight glare on highways, and can effectively improve the highway driving environment at night.

Many countries have introduced relevant standards for the height of anti-glare facilities. The existing standard in China stipulates that the height of the anti-glare facility should not exceed 2 m [10]. According to research conducted by the Transportation Research Board, on a flat and level divided highway without cross slope, glare screens would have to be the same height as the average driver’s eye, or 1.14 m, in accordance with AASHTO standards [11]. BSEN 12676-1:2000 gives the calculation formula for anti-glare facilities in the case of pavements with a constant longitudinal gradient [12]. The Guidelines for Expressways published by the Indian Roads Congress state that the height of a glare-reduction device should be set at 1.4–1.5 m on the assumption of combinations of opposing passenger vehicles and of a passenger car and a large vehicle moving in opposite directions [13]. Moreover, many scholars have also studied the height of anti-glare facilities on different freeway alignments. Liang used UC-win/Road simulation software to determine the acceptable height difference for drivers in the transition section; the results showed that the height difference should not exceed 6 cm when the radius of the concave vertical curve does not exceed 30,000 m [14]. Bagui recommended that the height of an anti-glare screen barrier should be 1.85 m in the Indian context [15]. Wu Yan put forward a calculation method for anti-glare plate heights on concave vertical curve sections. Research shows that when the lamp distance of car headlamps is 120 m and the radius of a concave vertical curve section is 12,000–32,000 m, the minimum design height of the anti-glare plate should be 1.72 m, and the maximum design height of the anti-glare plate should be 1.80 m [16]. Although different countries and scholars recommend different heights for anti-glare facilities, most of the existing studies calculate the height of anti-glare facilities by considering the height of the high-beam headlights of opposing vehicles, the height of the driver’s line of sight, the lateral distance from vehicles to anti-glare facilities, and the minimum effective height of anti-glare facilities from the road surface. Ma Yang [17] used a prismatic cone to simulate the spatial range variation of headlights in the actual driving process, and calculated the height of anti-glare facilities for any position. At present, the setting of anti-glare facilities mainly considers blocking disability glare; however, headlight leakage from the anti-glare facilities can still make drivers uncomfortable. This is not conducive to the driver’s identification of road obstacles, road conditions, and oncoming traffic conditions, and may cause traffic accidents. Therefore, it is crucial to study the illuminance thresholds and spatial distribution of glare perception caused by vehicle headlights on drivers.

The illuminance thresholds and spatial distribution of the glare from high-beam headlights on drivers fall under glare evaluation. Current research on glare focuses on both disability glare and discomfort glare. Disability glare is mainly evaluated by the threshold increment method [18,19]. Threshold increment (TI) is an evaluation index that expresses, as a percentage, the increase in the luminance contrast threshold required between an object and its background for it to be seen equally well with a source of glare present. The CIE 31:1976 report provides the formula [20], as shown in Equation (1), with $L_{v}$ and $\bar{L}$ in cd/m^2^ and for a range 0.05 < $\bar{L}$ < 5.

(1) $$TI=65\frac{L_{v}}{{\bar{L}}^{0.8}}\%$$

where $\bar{L}$ is the average road luminance and $L_{v}$ is the equivalent veiling luminance (cd/m^2^); $L_{v}$ is given by Equation (2):

(2) $$L_{v}=K\cdot \frac{E_{G1}}{\theta^{2}}$$

where K, for practical purposes, is taken as 10 when θ is expressed in degrees, or as $K=3\times 10^{-3}$ when θ is expressed in radians. For a total installation, the individual equivalent veiling luminances $L_{vi}$ of each luminaire have to be added together, as follows: $L_{v,total}=\sum_{i=1}^{n}L_{vi}$. $E_{G1}$ is the illuminance on the tested driver’s eye produced by the glare source in the plane perpendicular to the line of sight, in lux, while θ is the angle between the center of the glare source and the line of sight. The exponent of θ is valid from ~1.5° to 60° (0.025 to 1.00 radian).

However, it is impossible to guarantee driving safety if only disability glare is considered as the basis for the height of anti-glare facilities. Headlights can still interfere with drivers’ ability to recognize objects ahead and, thus, can still cause traffic accidents.

In the actual driving process, discomfort glare may divert the attention of drivers away from the scene to be surveyed towards the bright glare source [21]. However, not all discomfort glare will adversely affect driver safety, so it is crucial to classify discomfort glare, and to define the level of discomfort glare that is acceptable for drivers. The psychophysical method is a method that mainly evaluates the degree of discomfort glare, and the evaluation process is divided into three parts: (1) selection of representative subjects for subjective evaluation of uncomfortable feelings, (2) measurement of photometric parameters within the field of view, and (3) establishment of a correlation model between the subjective evaluation of feeling levels and photometric parameters [22]. Scholars have conducted many studies on the evaluation models of discomfort glare, and obtained evaluation models of the degree of discomfort glare for different scenes [23,24,25,26,27]. To describe perceptions of discomfort glare, various scholars have developed scales consisting of several words [25,26,28,29], among which the nine-point scale designed by de Boer et al. has been widely used [30]. However, some scholars have also raised different opinions to de Boer’s nine-point scale. Theeuwes et al. found that the commonly used de Boer glare scale is not suitable for testing driver performance [31]. There is no validated Chinese description of the de Boer scale; this causes differences in individual understanding of the Chinese meaning of the scale, leading to differences in test results [30].

In view of the above problems, this article proposes a subjective glare scale based on the analysis of the mechanism of glare from high-beam headlights. We study the threshold values and spatial distribution of glare perception at different longitudinal distances. The results can provide scientific evidence for calculating the reasonable heights of anti-glare facilities for expressways with various alignments.

## 2. Mechanism and Evaluation Method of Vehicle High-Beam Headlights Glare

Light with information about the illuminated object enters the subject’s eye through the pupil and is refracted by the crystalline lens, passing though the vitreous humor, and then falling on the retina. Light with information about the illuminated object is transmitted to the brain in the form of electronic impulse signals through the optic nerve, finally forming vision through a series of chemical reactions and transformations [32]. Moderate light provides environmental brightness and visual guidance for driving, but unreasonable light will have a negative impact on driving safety. Drivers need to complete the task of transportation and ensure the safety of driving during dynamic processes. For the particular characteristics of the driver, we propose the concept of traffic glare, which refers to the glare that results in the driver being unable to recognize the road information ahead and the surrounding environment during a given driving task because of visual discomfort or reduced visual ability.

**Figure 1:**
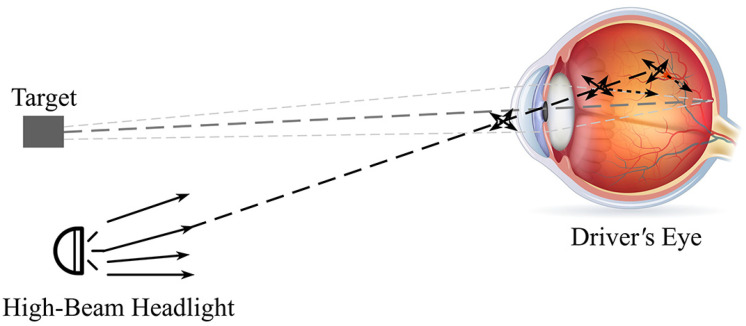
Light scatter in the eye due to high-beam headlight glare.

High-beam headlight glare is one form of traffic glare. High-beam headlight glare affects safe driving in two primary ways: (1) First, the intense light from high-beam headlights scatters in the opposite driver’s eye to form a bright veil, reducing retinal image contrast and, thus, reducing the overall visibility of objects laying ahead. Light scatter in the eye due to high-beam headlight glare is shown in Figure 1 [31]. Reduced visibility may affect the performance of visual tasks related to safe driving. (2) Second, the visual disturbance caused by the glare from high-beam headlights results in discomfort, and Berman et al. [33,34] concluded that discomfort glare effects coincide with uncomfortable contractions of the iris and the muscles surrounding the eyes.

To characterize the impact of high-beam headlight glare on driver safety, a combination of quantitative and qualitative methods was used to study the influence of opposing glare from vehicles’ high-beam headlights on drivers’ visual ability. The primary function of the vehicle headlamp is to provide a light environment for the driver to identify road traffic conditions within a certain distance, including information on the road ahead and the surrounding environment. According to the visual recognition requirements of the most unfavorable conditions, the criterion of traffic glare is whether the driver can find the gray target within a certain distance ahead in time under the interference of vehicle headlights. The 20 cm × 20 cm × 20 cm gray cube recommended by the Commission International de l’Eclairage is used as the visual target, and its surface reflection coefficient is 0.2.

The illuminance at the eye of the driver is the main factor affecting the glare experienced by the driver. To quantify the impact of high-beam headlights on the glare experienced by the driver, the vertical illuminance at the eye of the tested driver is measured. The existing glare research shows that both disability glare and discomfort glare are related to the relative position between the light source and the subject [22,35]. When the relative position between the driver and the high-beam headlights is different, the glare experienced by the driver is also different. The glare caused by the high-beam headlights will not always impact the driver’s ability to drive safely. When the included angle between the driver and the high-beam headlights is small or close, it is easy to produce disability glare, resulting in the driver’s inability to see the target in front within a short time, reducing driving safety. As the included angle between the driver and the high-beam headlights is enlarged or the distance increased, although the glare generated by the high-beam headlights is weakened, it still interferes with the driver’s recognition of the target ahead. Drivers want to avoid the glare by turning their heads, and traffic accidents can easily occur when the vehicle is moving at high speeds. When the angle between the driver and the high-beam headlights is further enlarged or the distance increased, although the driver will still feel the glare generated by the high-beam headlights, the glare experienced is weaker; the driver can recognize the target ahead, and can drive safely under these conditions. According to the level of glare experienced by the driver, the discomfort glare is classified into interference glare and acceptability glare. A subjective headlight glare scale and relevant description is shown in Table 1; according to the influence of glare on the driver’s vision, from strong to weak, it is divided into disability glare, interference glare, and acceptability glare. The degree of influence of different glare levels is described in combination with the test scene to facilitate the test driver’s distinguishing of different levels of glare.

The combination of quantitative measurements of vertical illuminance at the driver’s eye and subjective visual recognition evaluation of the tested driver was used to study the illuminance thresholds and the spatial distributions of disability glare–interference glare (DGIG) and interference glare–acceptability glare (IGAG) from high-beam headlights under different longitudinal distances. The results can provide a scientific basis for calculating the reasonable height of anti-glare facilities under different alignments.

## 3. Methodology

**Figure 2:**
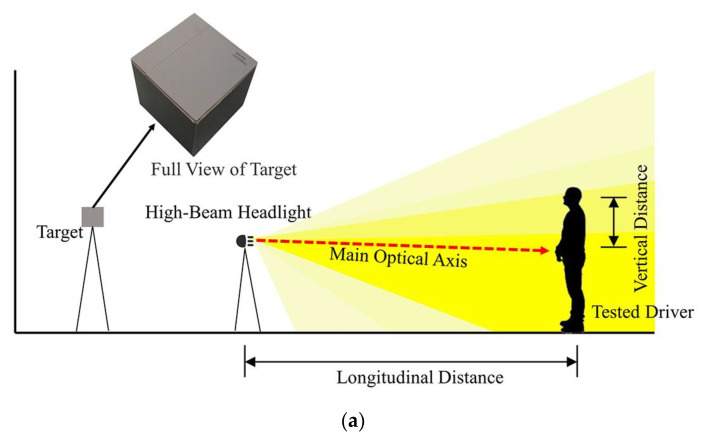

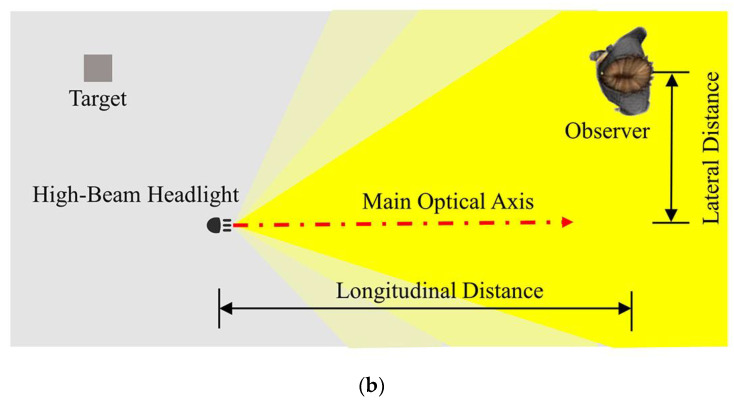
Diagram of the glare effect tests. (**a**) Side view, (**b**) Vertical view.

Glare effect tests collect the illuminance at the eye of tested drivers, along with the spatial distributions of DGIG and IGAG. The tests use longitudinal distance, lateral distance, and vertical distance to indicate the relative position between the high-beam headlights and the tested driver. A diagram of the glare effect tests is shown in Figure 2. The linear distance between the high-beam headlights and the tested driver is defined as the longitudinal distance. Lateral and vertical distances are measured upon the main optical axis of the headlights. The distance from the main optical axis of the headlights along the lateral direction is defined as the lateral distance. The distance from the main optical axis of the headlights along the vertical direction is defined as the vertical distance.

### 3.1. Laboratory

**Figure 3:**
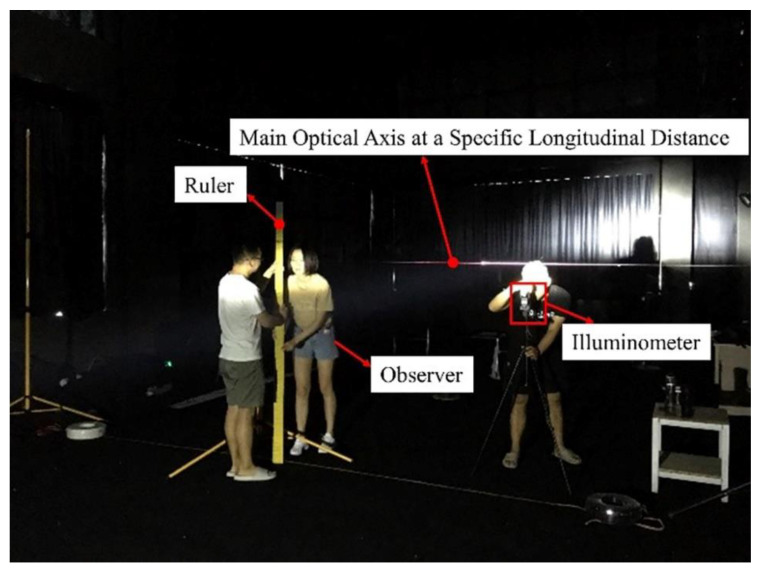
The laboratory.

Owing to a lack of artificial lighting at night on the expressway, the light environment mainly includes vehicle headlights and the Moon. Considering the most unfavorable working conditions of the driver’s safety recognition, we conducted the glare effect tests without background light in the environment. The size of the laboratory was 12 m long × 6 m wide. The laboratory is shown in Figure 3. The walls of the laboratory were covered with black cloth to reduce wall reflections. The ground was paved with reflective material close to the asphalt waterproof membrane. In order to make the driver see the target at the same angle, the tests used a variable height bracket to adjust the height of the target according to the position of the tested driver.

### 3.2. Light Source

The development of vehicle headlights has gone through four stages: incandescent bulbs, halogen lamps, high-intensity discharge (HID) lamps, and light-emitting diodes (LEDs). LED headlights are gradually becoming a major trend in vehicle headlights due to their long life, energy efficiency, fast response time, light weight and small size, high brightness, and wide spectral range [36,37]. Sivak [38] found that LED headlights containing more blue light were more likely to produce discomfort glare than HID headlights; this is because the spectral sensitivity of discomfort glare depends not only on the photopic vision or the combination of the spectral sensitivity of photopic vision and scotopic vision of the eyes, but also on the short-wavelength type of cone cells (so-called “S” or “blue-sensitive” cones) [39]. Moreover, in order to make LED headlights with sufficient luminous flux, multiple arrays of individual LED headlights are required, which means that the luminance distribution of the luminous surface of LED lights is non-uniform. Higashi [40] and Tashiro et al. [41] showed that the more non-uniform the luminance distribution of the luminous surface of the headlights, the more obvious the discomfort glare perception.

**Figure 4:**
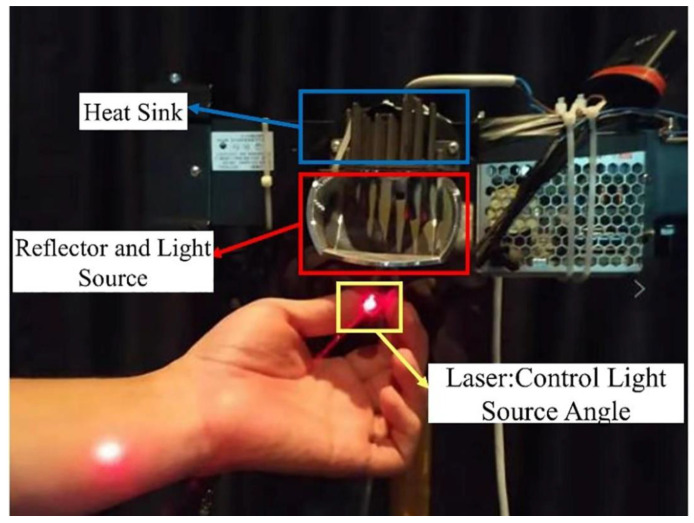
High-beam headlight binding.

Considering the most detrimental situation to the driver’s safety and the development trend of vehicle headlights, our test selected standard LED headlights in China. The high-beam highlight was fixed on a bracket with adjustable height and angle. A schematic diagram of the high-beam headlight binding is shown in Figure 4.

**Figure 5:**
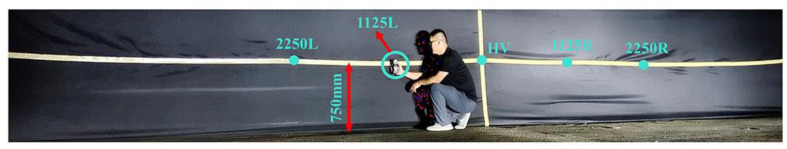
Field test diagram of the high-beam headlight.

**Figure 6:**
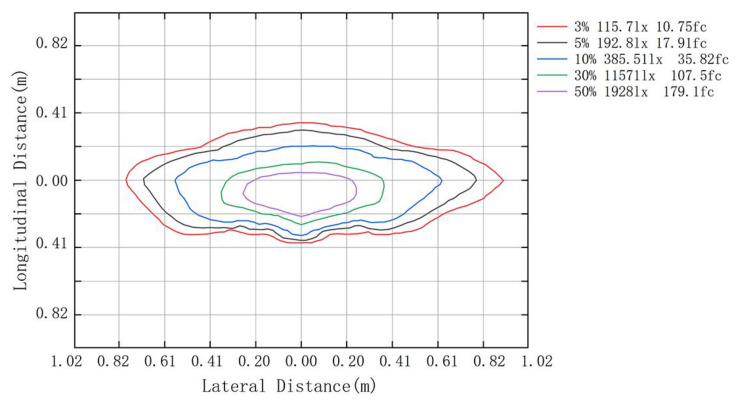
Isoilluminance diagram of the light source.

To verify the validity of the light source, the illuminance at HV, 1125L, 2250L, 1125R, and 2250R on the black cloth was measured at 25 m away from the light source. The field test diagram of the high-beam headlight is shown in Figure 5. The selected light source meets the requirements of “Automotive headlamps with LED light sources and/or LED modules (GB 25991-2010)” [42] for automotive headlamps through measurement. In order to obtain the optical characteristics of the high-beam headlight and guide the design of the test scheme, the parameters of the high-beam headlight were tested by integrating the sphere and goniophotometer. The maximum luminous intensity of the high-beam headlight was 97606cd, and the color temperature was 5853k. The isoilluminance diagram of the light source was measured with a goniophotometer. The installation height was 5 m due to limitations of the test equipment. The isoilluminance diagram is shown in Figure 6.

It can be seen from Figure 6 that the isoilluminance diagram of the high-beam headlight is approximately symmetrical on the left and right axes, and the illuminance values of the light source decrease sequentially from the center to the margins. The isoilluminance diagram is an inherent characteristic of the light source. The illuminance values and spatial distributions that cause the driver to experience different levels of glare need to be obtained through the glare effect test. To this end, we assumed that the spatial distribution of different glare perceptions for the tested drivers was similar to the isoilluminance diagram of the high-beam headlight, and took the right half for research. The longitudinal distances between the glare source and the tested driver were 3 m, 5 m, 7 m, 10 m, and 12 m, due to the limitation of the laboratory size.

### 3.3. Tested Drivers

According to the data published by The Ministry of Public Security of the People’s Republic of China, drivers aged 26–50 accounted for 70.71% of the total number of drivers by 2021. Therefore, in order to protect most drivers from the glare interference caused by the glare from headlights, our test took drivers aged 26–50 as participants. To avoid differential effects on the test results due to the gender, age, and personality of the tested drivers, 24 healthy drivers (mean = 36.8 years; standard deviation = 7.9 years) were randomly recruited. The subjects’ naked or corrected visual acuity had to be 4.9 or higher, and they had to be free of color blindness, weakness, or other eye diseases; they were banned from using alcohol or drugs during the glare effect tests.

### 3.4. Procedure

Glare effect tests use a combination of quantitative and qualitative methods to obtain the illuminance thresholds and spatial distributions of DGIG and IGAG with different longitudinal distances. The test drivers were numbered from No.1 to No.24.

The procedure was as follows:

(1) Install the headlamp and adjust the height of the target to the initial position. The height of the target changes continuously with the tested driver during the test;
(2) Train the driver to be familiar with the subjective headlight glare scale. Disability glare and interference glare are differentiated by whether the tested drivers want to turn their heads immediately to avoid the light. Interference glare and acceptability glare are differentiated by whether the tested drivers want to avoid the light and whether they can see the outline of the target ahead after the duration of visual recognition (1.5 s);
(3) Allow each of the tested drivers to fully adapt to the brightness of the background environment before the test.
(4) The tester uses the illuminometer to find the point with the maximum illuminance at the longitudinal distance of 3 m from the headlamp, and uses the laser to mark the height of this point along the lateral direction. In subsequent tests, the height of this point is used as the benchmark for relevant distance measurements;
(5) The 24 test drivers successively move to the right at the same height as the maximum illumination point to find the lateral apoapsis of DGIG. The tester measures and records the data. The tester analyzes the data to obtain the lateral apoapsis of DGIG. The distance between the origin and the lateral apoapsis is divided equally at an interval of 10–30 cm as the lateral observation point;
(6) Driver No.1 moves above the main optical axis of the first lateral observation point. When the interference glare perception appears, the tester measures the illuminance at the eye of the tested driver and the height difference between the eye position and the main optical axis;
(7) Drivers No.2–No.24 repeat step (6) above the main optical axis of the first lateral observation point. When the interference glare perception appears, the tester measures the illuminance at the eye of the tested driver and the height difference between the eye position and the main optical axis;
(8) The tested drivers repeat steps (6) and (7) above the main optical axis of the different lateral observation points. When the interference glare perception appears, the tester measures the illuminance at the eye of the tested driver and the height difference between the eye position and the main optical axis;
(9) The tested drivers repeat steps (6)–(8) below the main optical axis of the different lateral observation points. When the interference glare perception appears, the tester measures the illuminance at the eye of the tested driver and the height difference between the eye position and the main optical axis;
(10) The tested drivers repeat steps (5)–(9) in the upper and lower sides of the main optical axis of the different lateral observation points. When the acceptability glare perception appears, the tester measures the illuminance at the eye of the tested driver and the height difference between the eye position and the main optical axis;
(11) The tested drivers repeat steps (4)–(10) to complete the corresponding test content and collect relevant data when the longitudinal distance is 5 m, 7 m, 10 m, and 12 m.

### 3.5. Analysis Method

#### 3.5.1. Photometric Inverse Square Law

**Figure 7:**
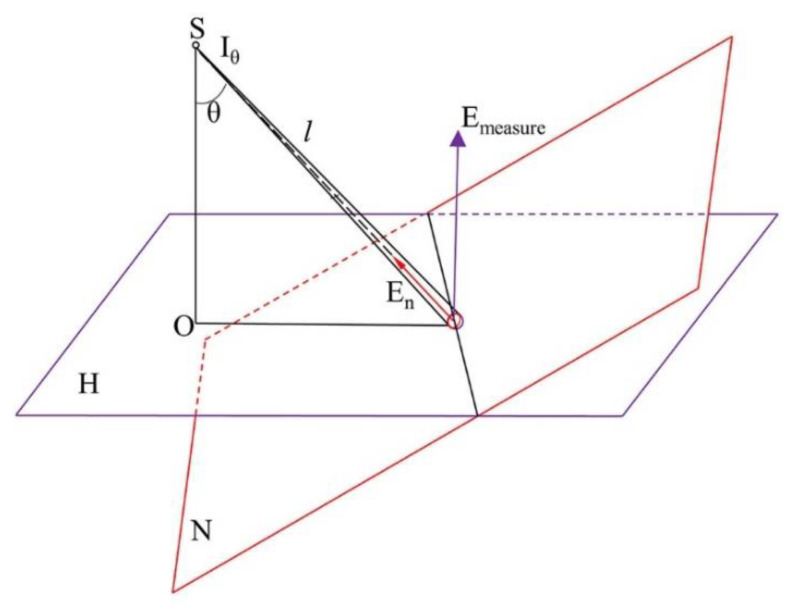
Conversion diagram of vertical illuminance of the tested driver’s eye and the pointing plane illuminance.

Because there is a certain angle between the tested drivers and the light source, it is necessary to convert the vertical illuminance of the tested driver’s eye $E_{measure}$ into the pointing plane illuminance $E_{n}$. The conversion diagram of vertical illuminance of the tested driver’s eye and pointing plane illuminance is shown in Figure 7. Equations (3) and (4) relate to the conversion formula between illuminance at the driver’s eye and pointing plane illuminance within 120 m, and the calculation formula of luminous intensity that makes the tested driver feel glare, respectively.

(3) $$E_{n}=\frac{E_{measure}}{\cos\theta }$$

(4) $$I_{\theta }=E_{n}\cdot l^{2}$$

where $E_{n}$ is the pointing plane illuminance; $E_{measure}$ is the vertical illuminance of the tested driver’s eye; θ is the angle between the direction of the point light source S pointing towards the illuminated point *p* and the direction of the lead hammer; $I_{\theta }$ is the luminous intensity that produces the perception of glare; and l is the distance between the glare source and the tested driver’s eyes.

#### 3.5.2. Ellipse Fitting Equation

The ellipse fitting equation is shown in Equation (5):

(5) $$\left\{ \begin{array}{l} \frac{x_{i}^{2}}{a_{i}^{2}}+\frac{y_{i}^{2}}{b_{i}^{2}}=1\\ a_{i}=k_{i}\cdot i+c_{i}\\ b_{i}=k_{i}\cdot i+d_{i} \end{array}\right.$$

where when the curve lies in the upper half of the *x*-axis, $0\leq x_{i}\leq a_{i}$, $0\leq y_{i}\leq b_{i}$; when the curve lies in the lower half of the *x*-axis, $0\leq x_{i}\leq a_{i}$, $b_{i}\leq y_{i}\leq 0$; $a_{i}$ is the maximum lateral distance at a longitudinal distance of i m; $b_{i}$ is the maximum or minimum vertical distance at a longitudinal distance of i m; i is the longitudinal distance (m); and $k_{i}$, $c_{i}$, and $d_{i}$ are constants at a different longitudinal distances.

## 4. Results and Analysis

When the longitudinal distance is constant, the illuminance at the eye of the tested drivers is related to the lateral distance and vertical distance, and has a one-to-one correspondence with the vertical distance. Therefore, the determination of the values of the lateral distance and vertical distance is very important. Due to the individual visual recognition difference in the test, in order to ensure that the obtained lateral apoapsis and vertical distance could meet the visual recognition needs of most drivers for safety and comfort, the test results of drivers with a cumulative frequency of 85% were used as the lateral apoapsis and vertical distance at the borderline of DGIG and IGAG. During the test, if there was no illuminance value for the vertical distance corresponding to the calculated 85% quantile, the tester supplemented the illuminance value at this position.

### 4.1. Distribution of Lateral Apoapsis

**Figure 8:**
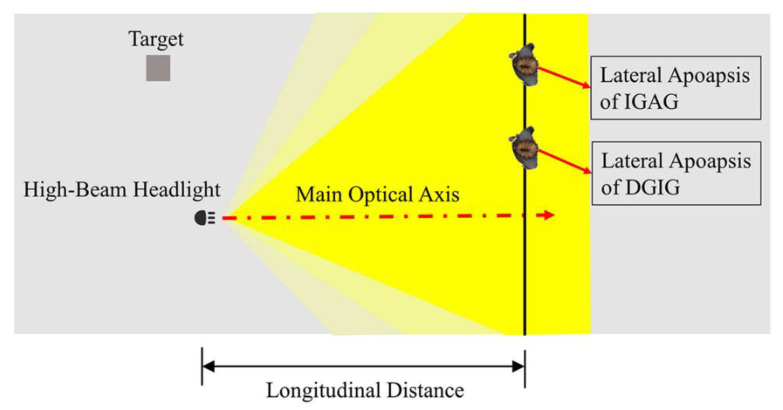
Diagram of lateral apoapsis (vertical distance = 0).

**Figure 9:**
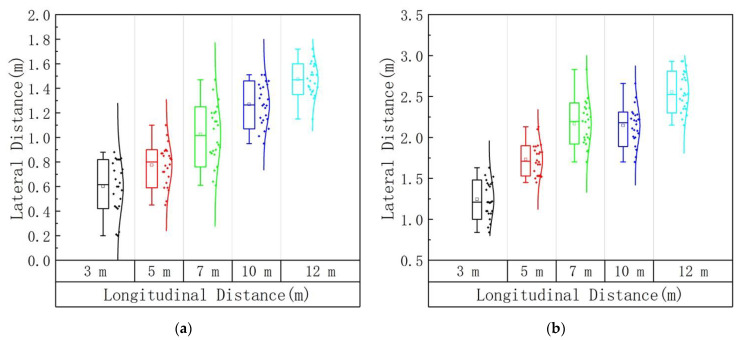
Lateral apoapsis under different longitudinal distances: (**a**) DGIG; (**b**) IGAG.

The lateral apoapsis at the borderline of the DGIG and the IGAG was obtained from the glare effect test at the longitudinal distances of 3 m, 5 m, 7 m, 10 m, and 12 m. The schematic diagram of the lateral apoapsis is shown in Figure 8. The distribution of lateral apoapsis under different longitudinal distances is shown in Figure 9. It can be seen from the figure that the distribution of the lateral apoapsis under different longitudinal distances is relatively concentrated, and essentially conforms to the normal distribution. To account for age differences between the tested drivers, we performed a one-way analysis of variance on their lateral apoapsis. A repeated-measures AVONA on the data demonstrated that age has no significant effect on the lateral apoapsis. The confidence interval of the variance analysis was 95%. The difference analysis results regarding age and lateral apoapsis under different longitudinal distances are shown in Table 2.

The values of the lateral apoapsis corresponding to different glare perceptions at different longitudinal distances are shown in Table 3. It can be seen from Table 3 that the lateral apoapsis at the borderline of the DGIG is less than the lateral apoapsis at the borderline of the IGAG. The general trend is that the lateral apoapsis increases with the increase in the longitudinal distance.

### 4.2. Range of Glare Effects at Different Longitudinal Distances

**Figure 10:**
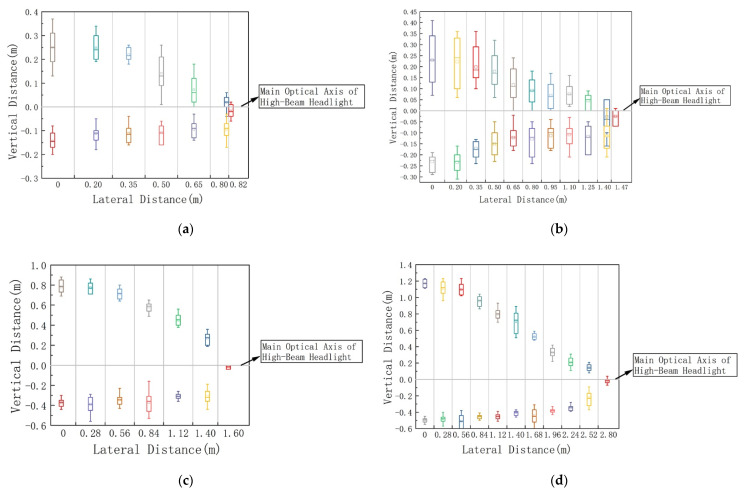
Statistical boxplots of vertical distance under different lateral distances: (**a**) DGIG (longitudinal distance = 3 m); (**b**) IGAG (longitudinal distance = 3 m); (**c**) DGIG (longitudinal distance = 12 m); (**d**) IGAG (longitudinal distance = 12 m).

Taking the longitudinal distances of 3 m and 12 m as examples, the vertical distance distribution on the cutoff curves of DGIG and IGAG is shown in Figure 10. The colors in the figure represent the vertical distance distribution at different lateral distances. The vertical distance is divided into two parts by the main optical axis of the high-beam headlights; therefore, each lateral distance corresponds to two vertical distances. This shows that the vertical distance is the largest when the lateral distance is equal to 0 m, and the vertical distance decreases with the increase in lateral distance. In order to verify the difference in age on the vertical distance that produces the same glare perception, we performed a one-way analysis of variance on their vertical distance. The confidence interval of the variance analysis was 95%. The difference analysis results regarding age and vertical distance under different longitudinal distances are shown in Table 4. A repeated-measures AVONA on the data demonstrated that age has no significant effect on vertical distance.

**Figure 11:**
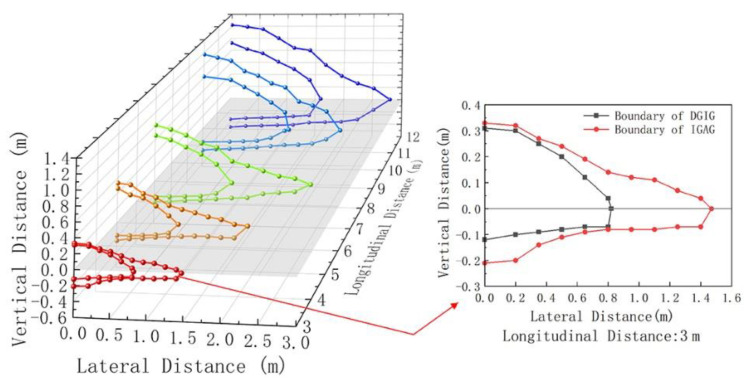
Cutoff curves of DGIG and IGAG.

The cutoff curves of DGIG and IGAG under different longitudinal distances are shown in Figure 11. As shown in Figure 11, the closer the lateral observation position of the driver to the main optical axis of the glare source, the stronger the subjective perception of glare. As the lateral and vertical distances increase, the disability glare gradually decreases to interference glare, and then to acceptability glare. The range of IGAG contains the range of DGIG. Comparing the range of glare effects under different longitudinal distances shows that the range of DGIG and IGAG tends to increase as the longitudinal distance increases.

### 4.3. Illuminance Thresholds of the Driver’s Eye at Different Longitudinal Distances

**Figure 12:**
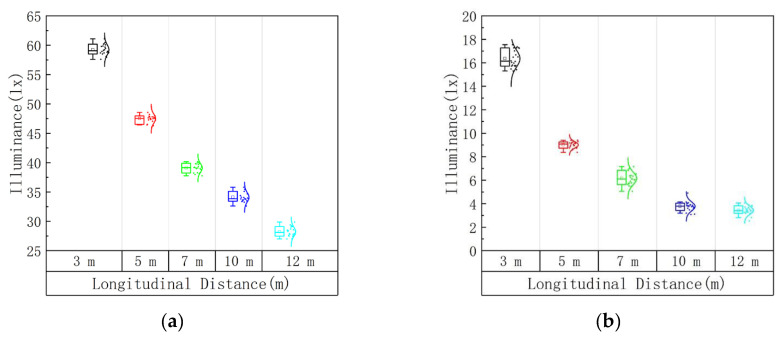
Distribution of illuminance thresholds under different longitudinal distances: (**a**) DGIG; (**b**) IGAG.

The illuminance at the eye of tested drivers has a one-to-one correspondence with the vertical distance. The difference analysis results in Section 4.2 regarding age and vertical distance under different longitudinal distances show that age has no significant effect on vertical distance; therefore, age also has no significant effect on the illuminance at the eye of the driver. The distribution of illuminance thresholds of different glare levels at different longitudinal distances is shown in Figure 12. The illuminance threshold values of DGIG and IGAG at different longitudinal distances are shown in Table 5. The data of the figure and table show that the illuminance thresholds of IGAG tend to decrease as the longitudinal distance increases at different longitudinal distances. The vertical illuminance at the driver’s eye on the cutoff lines of DGIG and IGAG under the same longitudinal distance is almost equal.

The Design Guidelines for Highway Safety Facilities (JTG D81-2017) [10] give the irradiation distance of high-beam headlamps as generally ~120 m, and stipulate that 120 m is used to calculate the glare distance of anti-glare facilities. It is for this reason that obtaining the illuminance thresholds at different longitudinal distances within 120 m is essential. The value of luminous intensity causing the glare perception of the tested driver was calculated through the photometric inverse square law, as shown in Section 3.5.1. By fitting the correlation between the longitudinal distance and the corresponding luminous intensity, a linear relationship between longitudinal distance and luminous intensity was found. The illuminance thresholds for different longitudinal distances were deduced from this law. Verification by outdoor data at 50 m and 100 m shows that the calculated results were consistent with the actual situation. The illuminance thresholds of DGIG and IGAG for different longitudinal distances are shown in Table 6.

### 4.4. Spatial Distribution

**Figure 13:**
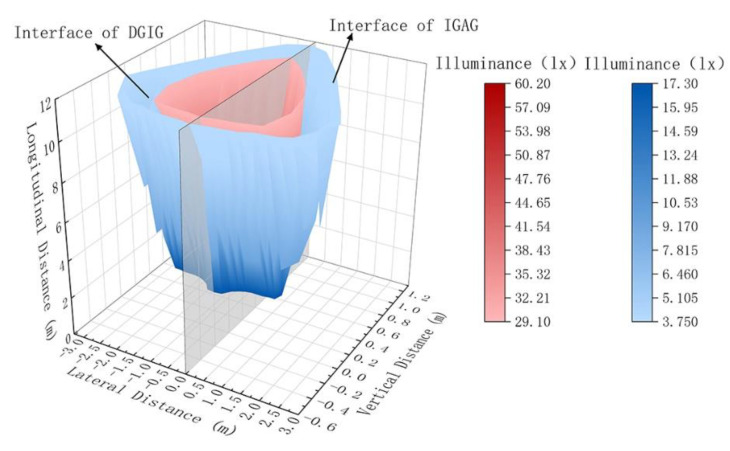
Spatial distribution of glare at the longitudinal distance of 3 m to 12 m.

Experimental studies have shown boundaries to the spatial distribution that make drivers experience different levels of glare. The spatial distribution of glare at the longitudinal distance of 3 m to 12 m is shown in Figure 13.

In order to obtain the spatial distribution of glare at different longitudinal distances within 120 m, the measured data need to be extrapolated. As can be seen from Figure 11 and Figure 13, the shape of the spatial distribution of glare is close to pyramid-like, with an ellipse as the cross-section, which is assumed to be pyramid-like with a regular ellipse as the cross-section to fit the cutoff lines of DGIG and IGAG.

**Figure 14:**
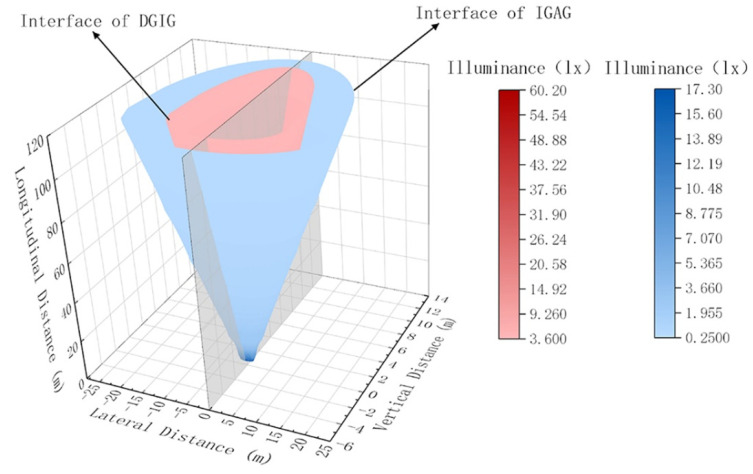
Spatial distribution of DGIG and IGAG at different longitudinal distances within 120 m.

According to Equation (5), the spatial distribution of DGIG and IGAG can be calculated within 120 m. The spatial distribution of DGIG and IGAG at different longitudinal distances is shown in Figure 14.

## 5. Discussion

The cutoff curves of DGIG and IGAG (as shown in Figure 11) were obtained through the glare effect test. The assumption that the distribution diagrams of different glare levels of the tested drivers are similar to the isoilluminance diagram of high-beam headlights is correct. Figure 11 shows that the glare level of the tested drivers was reduced from disability glare to interference glare, and then to acceptability glare, with the increase in lateral distance or vertical distance at the same longitudinal distance. The main reason for this is that the angle between the main optical axis of the headlights and the tested driver’s line of sight increases with the change in the vertical or lateral distance between the tested driver and the headlights; the light entering the tested driver’s eyes is thus reduced, which reduces the glare experienced by the tested driver.

It can be seen from Figure 12 that the vertical illuminance thresholds of DGIG and IGAG are almost equal under the same longitudinal distance. Table 5 shows that the vertical illuminance thresholds decrease with the increase in longitudinal distance. This may be because the light source not only provides the required light environment for the test, but also provides the background light environment. However, with the increasing longitudinal distance between the tested drivers and the light source, the gradient of the background luminance generated by the light source decreases more quickly. When the background luminance decreases, it is easy to form luminance contrast in the eyes of the tested driver, making it more likely to produce disability glare or interference glare.

In existing studies, the height design of anti-glare facilities primarily takes into account the height of the driver’s line of sight and that of the high-beam headlights, as well as lane width and road alignment [10,12,43,44]. Ma Yang [17] used a prismatic cone with the center of the headlight line as the vertex of the vehicle light irradiation range as the basis for calculating the height of anti-glare facilities. Both of these methods have shortcomings. On the one hand, the influence of headlights is calculated by a ray of light only, which may lead to light leakage in the special linear road section. On the other hand, the entire influence range of headlights is included in the calculation of the height of anti-glare facilities, which may lead to the excessive height of said facilities, as the highway anti-glare facilities should be adjusted in light of not only their anti-glare effect, but also the effect of wind load on them [43]. If the anti-glare facilities are too high, they may be unsafe due to the wind load. It was found through the glare effect tests that the entire irradiation range of headlights does not have an impact on driver safety. The spatial distribution of the high-beam headlight on the driver is a pyramid-like shape. Parameters such as the relative distance between high-beam headlights and the driver, the width of the driving lane, driver-sight height, and the height of the headlamp were considered, and the models of the height of the anti-glare facilities as a function of the most unfavorable angle for expressways with different alignments were put forward. The height of the anti-glare facilities determined by the calculation method can meet the visual recognition requirements of driving safety, and the safety of highway driving at night can be improved.

## 6. Limitations and Directions for Future Research

The main limitation of this study was the age of the tested drivers. The ages of tested drivers ranged from 26 to 50 years old, and elderly drivers were not included. Previous studies have shown that elderly drivers are more sensitive to glare effects [45]. This issue will be investigated based on the need for visual recognition requirements of elderly drivers in future research.

## 7. Conclusions

Based on the drivers’ visual recognition requirements, the glare effect tests were conducted to study the illuminance thresholds and spatial distributions that cause different glare perceptions of drivers at different longitudinal distances. The main conclusions to be drawn from this analysis are as follows:

(1) The illuminance thresholds of glare that cause the same glare perception of the subject driver are almost equal within the same longitudinal section of the light source;
(2) As lateral distance increases, the disability glare gradually decreases to interference glare, and then to acceptability glare, and the vertical distance also tends to decrease in the same longitudinal section of the light source;
(3) As the longitudinal distance of the light source increases, the illuminance thresholds of DGIG and IGAG tend to decrease;
(4) With the increase in the longitudinal distance of the light source, the spatial distribution of glare gradually becomes larger. The spatial distribution of glare can be combined with the height of the driver’s sight, the height of the headlights, the width of the carriageway, and the road alignment to provide scientific evidence for calculating the reasonable heights of anti-glare facilities for expressways with different alignments.

## Figures and Tables

**Table 1:** Subjective headlight glare scale and relevant description.

Glare Level	Description
Disability glare	Intense feeling of glare, serious blinding, difficult to open eyes, cannot see the target
Interference glare	Slightly stronger glare feeling, dazzling, the eyes want to avoid the line of sight, can recognize the target, the outline is not clear
Acceptability glare	Slightly blinding, visual recognition is essentially unaffected by light, the visual recognition of the target is essentially unaffected, the outline is clearer

**Table 2:** Difference analysis results regarding age and lateral apoapsis under different longitudinal distances.

Longitudinal Distance	DGIG		IGAG	
	F	*p*	F	*p*
3 m	0.607	0.807	0.608	0.806
5 m	1.024	0.519	1.325	0.368
7 m	0.737	0.712	0.394	0.942
10 m	1.740	0.234	0.535	0.858
12 m	1.276	0.390	0.452	0.911

**Table 3:** Values of the lateral apoapsis corresponding to different glare perceptions at different longitudinal distances.

Longitudinal Distance (m)	DGIG	IGAG
3	0.82	1.47
5	0.90	1.90
7	1.20	2.40
10	1.45	2.30
12	1.60	2.80

**Table 4:** Difference analysis results regarding age and vertical distance under different longitudinal distances.

Longitudinal Distance	DGIG			IGAG		
	SD	F	*p*	SD	F	*p*
3 m	0.0216	2.115	0.16	0.044	2.767	0.088
5 m	0.033	2.44	0.12	0.0423	2.962	0.076
7 m	0.0522	3.08	0.061	0.0289	1.989	0.181
10 m	0.0364	2.11	0.269	0.0318	2.832	0.074
12 m	0.034	3.17	0.064	0.071	3.07	0.069

**Table 5:** Illuminance thresholds of DGIG and IGAG at different longitudinal distances (0–12 m).

Longitudinal Distance (m)	DGIG			IGAG		
	Mean	Standard Deviation	P85	Mean	Standard Deviation	P85
3	59.22	0.879	60.16	16.36	0.732	17.28
5	47.50	0.762	47.96	9.01	0.274	9.25
7	39.10	0.729	39.89	6.16	0.537	6.80
10	34.10	0.796	35.05	3.77	0.390	4.04
12	28.22	0.777	29.10	3.44	0.343	3.80

**Table 6:** Illuminance threshold values of DGIG and IGAG for different longitudinal distances (0–120 m).

Longitudinal Distance (m)	Illuminance Thresholds of DGIG (lx)	Illuminance Thresholds of IGAG (lx)
3	60.16	17.28
5	47.96	9.25
7	39.89	6.80
10	35.05	4.04
12	29.10	3.80
15	25.85	2.54
20	20.32	1.85
25	16.71	1.45
⋮⋮	⋮⋮	⋮⋮
105	4.30	0.32
110	4.11	0.31
115	3.94	0.29
120	3.78	0.28

## Data Availability

Data generated in this study are available upon request.

## Abbreviations

Disability glare–interference glare	DGIG
Interference glare–acceptability glare	IGAG
High-intensity discharge lamp	HID
Light-emitting diode	LED
